# Acute angle closure triggered by oral benzodiazepines

**DOI:** 10.5935/0004-2749.20210017

**Published:** 2025-02-02

**Authors:** Alexis Galeno Matos, Pedro Duarte Barreto Castillo, João Augusto Lima Bisneto, Jayter Silva Paula

**Affiliations:** 1 Fundação Leiria de Andrade, Fortaleza, CE, Brazil; 2 Centro Universitário Christus, Fortaleza, CE, Brazil; 3 Department of Ophthalmology, Otorhinolaryngology, and Head and Neck Surgery, Faculdade de Medicina de Ribeirão Preto, Universidade de São Paulo, Ribeirão Preto, SP, Brazil

**Keywords:** Benzodiazepines/adverse effects, Intraocular pressure, Glaucoma, angle-closure/chemically induced, Iridectomy, Lasers, Benzodiazepinas/efeitos adversos, Glaucoma de ângulo fechado/ induzido quimicamente, Iridectomia, Lasers

## Abstract

Benzodiazepines are psychoactive drugs that are prescribed worldwide with limited information on their ocular side effects. Acute angle closure glaucoma is an adverse event with a high risk of blinding, especially in the elderly. We report two patients under 45 years old who presented with bilateral acute angle closure secondary to use of two long half-life benzodiazepines (clonazepam and alprazolam). In addition to suspending the use of these medications and administering ocular hypotensive drugs, both patients were successfully treated with bilateral peripheral laser iridotomy. To the best of our knowledge, this is the first report of bilateral acute angle closure secondary to the use of clonazepam and alprazolam.

## Abstract (pt)

Os benzodiazepínicos são medicamentos psicoati vos prescritos em todo o mundo, mas com poucas informa ções sobre seus efeitos colaterais oculares. O glaucoma por fechamento agudo do ângulo iridocorneano é um dos eventos adversos com maior risco de cegueira, sendo descrito particularmente em idosos. Relatamos aqui dois pacientes com menos de 45 anos de idade, com fechamento agudo do ângulo bilateral secundário ao uso de dois diferentes benzodiazepínicos de meia-vida longa (clonazepam e alprazolam). Além da suspensão dessas medicações e do tratamento clínico com drogas hipotensoras oculares, ambos os casos alcançaram sucesso com iridotomias periféricas bilaterais à laser. Considerando o conhecimento atual, estes são os primeiros relatos de fechamento agudo do ângulo bilateral secundária ao uso de clonazepam e alprazolam.

## INTRODUCTION

About 26% of people over 18 years old in the United States present with psychiatric conditions. In the elderly, anxiety disorder, severe cognitive impairment, and mood disorders (including bipolar disorder or depression) are the most common mental diseases. Ten percent of older adults use at least one antidepressant drug^(1)^.

Ocular adverse effects associated with antidepressant medications have been substantially reported in the literature. Recent studies have shown an association between antidepressant treatment and the development of acute angle closure (AAC)^(2)^, which leads to a rapid elevation of intraocular pressure (IOP). Risk factors associated with primary AAC include family history, advanced age, female gender, Asian ancestry, low anterior chamber depth, lens vault, anterior vault, and narrow iridocorneal angle associated with short axial length^(3)^.

The proposed explanation for AAC associated with psychiatric drugs may involve multifactorial conditions and two mechanisms of action. Edema and rotation of the ciliary body, relaxation of the zonula, and anteriorization of the lens were observed in patients with AAC associated with probable uveal effusion. These findings have been related to the use of topiramate, a widely used psychiatric drug indicated for the treatment of epilepsy, migraine, alcohol and nicotine addiction, peripheral neuropathy, and posttraumatic stress^(2)^. Zelefsky et al. reported a case of bilateral uveal effusion secondary to angular closure attributable to a selective serotonin reuptake inhibitor^(4)^, similar to a reported case of a patient using topiramate^(5)^. The second proposed mechanism of action for AAC is mydriasis caused by central and peripheral acetylcholine blockers, which may trigger AAC in predisposed narrow-angle eyes. This effect has been described for specific tricyclic antidepressants, selective serotonin reuptake inhibitors, and benzodiazepines^(5)^.

Benzodiazepines are mainly used for the treatment of anxiety disorder, depression, insomnia, alcohol dependence, and epilepsy. They are also used to produce muscle relaxation^(6)^. They are widely distributed in the body tissues, preferentially in lipid-rich areas such as the central nervous system and adipose tissue. The chemical structure of each drug influences penetration of ocular tissue, and therefore the degree of ocular action may vary among drugs. Benzodiazepines can be classified based on their half-life or onset of action^(7)^.

The ocular effects of benzodiazepines are diverse and include eye movement changes and altered contrast sensitivity^(5)^. Bilateral angle closure is observed mostly in the elderly^(6)^. We report here for the first time the cases of two nonelderly patients presenting with bilateral AAC after a single use of two long half-life benzodiazepines, clonazepam and alprazolam.

### Case 1

A 44-year-old white man was referred to an ophthalmological emergency unit with pain and blurred vision in OS after taking a single clonazepam 0.25 mg drop due to an anxiety attack 24 hours previously. Although no family history of glaucoma was reported, he confirmed a diagnosis of generalized anxiety disorder and alcohol and cigarette addiction. On examination, his OS presented conjunctival hyperemia and moderate corneal edema, with mydriasis and a shallow anterior chamber. Gonioscopy was not possible due to the corneal edema. Goldmann tonometry IOP was 28 mmHg OD and 54 mmHg OS, and no other ocular change was observed in OD. IOP was controlled after the use of oral acetazolamide 250 mg QID, topical prednisolone acetate 1% six times per day, and a topical combination of brimonidine tartarate 0.2% and timolol maleate 0.5% BID in OU. He had a refractive error of + 4.5 DS/-1.5 DC x 180^o^ in OD and + 4.0 DS/-1.5 DC x 170^o^ in OS. Axial length measurements were 20.65 mm OD and 20.67 mm OS. Gonioscopy revealed closed angles in 360 degrees, with a partial opening (posterior trabecular meshwork visible) after indentation in two quadrants without any synechiae in OU. Bilateral fundus examination revealed healthy optic nerve heads with vertical cup-to-disc ratios of 0.5. No perimetric examfination was performed.

**Figure 1:**
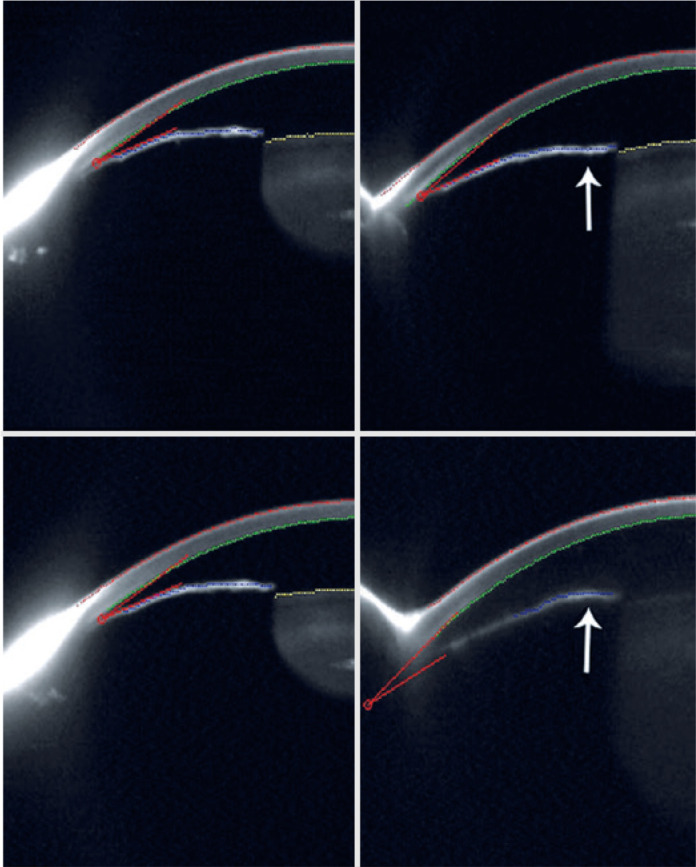
Scheimpflug image of the 3D-scan Oculus Pentacam illustrating the low volume of the anterior chamber before peripheral laser iridotomies, in the first upper image the right eye (superior angle 19.3^o^) and next to the left eye (inferior angle 25.7^o^) after a provocative test in a dark room with a shallow anterior chamber in relation to the inferior images, first referring to the right eye (superior angle 20.2^o^) and the left eye (inferior angle 21.2^o^) side, obtained days after the resolution of the acute angle closure crisis. In the left eye, the effect of pilocarpine changed the shape of the iris collar (arrow).

After receiving a topical combination of brimonidine tartarate 0.2% and timolol maleate 0.5% BID in OU asso ciated with 1% pilocarpine TID in OS, the patient underwent a provocative test in a dark room, and IOP in OD increased by 8 mmHg. He then underwent peripheral laser iridotomy in both eyes (Figure 1).

Interestingly, he reported a 3-month period of using oral bromazepam 6 mg per day for the treatment of anxiety attacks with mild sporadic, transitory discomfort in OS over the past 6 months. He also described occasional use of midazolam and zolpidem without any ocular symptoms in the past.

### Case 2

A 37-year-old white man was referred to an ophthalmological emergency unit reporting pain and blurred vision in the right eye after taking alprazolam 0.5 mg on the previous three nights to aid in the onset of sleep. No comorbidities, family history of glaucoma, or eye complaints were reported. On examination, he presented conjunctival hyperemia, mydriasis, a shallow anterior chamber, and moderate corneal edema that impaired the gonioscopic examination in OU. Goldmann tonometry IOP was 66 mmHg OD and 34 mmHg OS.

IOP was controlled after the use of oral acetazolamide 250 mg QID, topical prednisolone acetate 1% six times a day, and a topical combination of brimonidine tartarate 0.2% and timolol maleate 0.5% BID in OU. He had a refractive error of + 2.5 DS/-1.25 DC × 15^o^ in OD and + 3.50 DS/-3.5 DC × 25^o^ in OS. Axial length measurements were 21.64 mm OD and 21.38 mm OS. Gonioscopy revealed closed angles in 360 degrees OU, with partial opening (posterior trabecular meshwork visible) in the four quadrants in OD and complete opening (scleral spur visible) in the four quadrants in OS after indentation, without any synechiae. Bilateral fundus examination revealed healthy optic nerve heads with cup-to-disc ratios of 0.4. No perimetric examination was performed.

**Figure 2:**
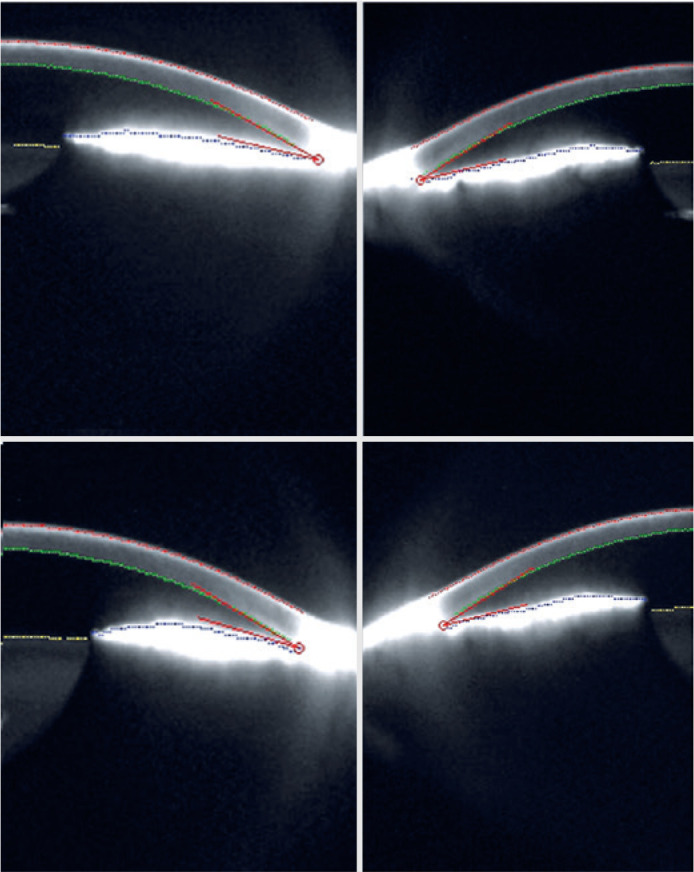
Scheimpflug image of the 3D scanning Oculus Pentacam illustrating the low anterior chamber volume of both eyes before peripheral laser iridotomies. In the first upper image, the right eye (inferior angle 27.1^o^) and the left side (inferior angle 28.5^o^) after treatment of angular closure and below, the right eye (inferior angle 22.2^o^) and the left side (inferior angle 26.7^o^) after provocative test showing right eye iris in mydriasis and anteriorization of the lens.

During the use of a topical combination of brimonidine tartarate 0.2% and timolol maleate 0.5% BID OU, the patient was invited to undergo a provocative test in a dark room. After the test, IOP increased by 10 mmHg OD and 8 mmHg OS, and peripheral laser iridotomies were successfully performed in OU (Figure 2).

## DISCUSSION

Although several drugs are known to cause anterior positioning of the iris-ciliary complex, even in myopic patients, antidepressant and anxiolytic agents are more likely to precipitate AAC^(2)^.

To the best of our knowledge, this is the first report of the occurrence of bilateral AAC secondary to the use of clonazepam and alprazolam in two adult patients. No previous reports have described AAC episodes after benzodiazepine use in patients under 60 years old. We speculate that the combination of a higher frequency of both benzodiazepine use and cataract in this age group may be a risk factor for angular narrowing and facilitate AAC. It is noteworthy that neither patient had used other medications with a potential risk of AAC. However, concomitant use of potential angular closure-inducing drugs has not been significantly associated with the risk of AAC^(6)^.

Regarding the mechanism of action in the central ner vous system, topiramate modulates different ion channels and enhances GABA inhibition. Benzodiazepines, tricyclic antidepressants, and selective inhibitors of neuronal uptake of either serotonin (e.g., sertraline) or both serotonin and norepinephrine (e.g., venlafaxine) contribute to the blockage of the neurotransmitter acetylcholine in the central nervous system and may interfere with the parasympathetic tonus of the iris and ciliary body muscles^(5)^. In a large survey including 13.5 million adult users of benzodiazepines, very few patients presented higher IOP. AAC triggered by benzodiazepines has been reported in elderly patients^(6)^. In fact, a population-based study has shown a low association between benzodiazepine use and AAC in patients under 60 years old. The authors also reported that the risk of AAC was associated with the use of benzodiazepines in people who had not been previously exposed to these drugs. First-time users have an increased risk of AAC, particularly within the initial 7 days after benzodiazepine ingestion^(6)^. However, this study was conducted in Korea, and ethnic differences in anatomical features may have influenced the observed risks.

Perera et al. reported a case of bilateral AAC in a 71-year-old woman on the sixth day after nitrazepam use^(8)^, and other authors have also reported bilateral AAC with the use of sertraline and venlafaxine^(2,9)^. Both of our patients presented with AAC within 12 hours after taking benzodiazepine tablets. However, patient 1 was not a first user, since he had been taking other benzodiazepines (midazolam and bromazepam) over the past 6 months, with minor signs of ocular changes only during the 3-month period of bromazepam use. We speculate that he did not experience ocular changes with midazolam because of its pharmacokinetic properties. Due to its shorter half-life and more rapid nervous central system penetration compared with other benzodiazepines, midazolam might not cause sufficient pupil dilation to increase the risk of AAC. Similarly, Park et al. reported no risk of AAC report that lipid solubility and protein binding affinity are factors that influence ocular tissue penetration and action of benzodiazepines^(6)^. Furthermore, an increased neural adrenergic tone associated with anxiety observed in this condition may also have acted as a triggering factor.

We are aware that both eyes of the patients reported here might have narrow angles and would be classified as suspected primary angle closure eyes. Gonioscopic findings, axial length, and refraction account to such diagnosis, as well as the positive head-down test in a dark room, even with its low predictive diagnostic value, which was observed in all eyes except the one under pilocarpine treatment^(10)^. These anatomical conditions are related to the risk of AAC, which was triggered by benzodiazepine use^(5)^. Both cases were considered successfully treated with bilateral peripheral laser iridotomy, which may prevent further AAC in these eyes. We are aware that in most cases of benzodiazepine-associated AAC, suspension of the triggering medication, along with the use of oral acetazolamide, topical hypotensive drops, corticosteroids, and cycloplegic agents, may resolve this clinical condition.

Many general physicians are not aware of or tend to ignore the risk of ocular adverse effects when prescribing psychiatric medications. All patients older than 60 years, as well as patients presenting with hypermetropia or any history suspicious of glaucoma, should be examined by an ophthalmologist before beginning long-term anticholinergic drug therapy. Patients, especially the elderly, should be advised of possible symptoms arising from increased IOP and should be monitored for visual disturbances during the initial period after beginning to use benzodiazepines.
